# Supplementary material for: Limited HIV-1 Subtype C nef 3′PPT Variation in Combination Antiretroviral Therapy Naïve and Experienced People Living with HIV in Botswana
Source: Pathogens. 2021 Aug 13;10(8):1027. doi: 10.3390/pathogens10081027 (PMC8400509; doi:10.3390/pathogens10081027)
Supplement: Supplementary file 1 [file pathogens-10-01027-s001.zip › supplementary Materials_13Aug2021_kks.pptx]

## Slide 1
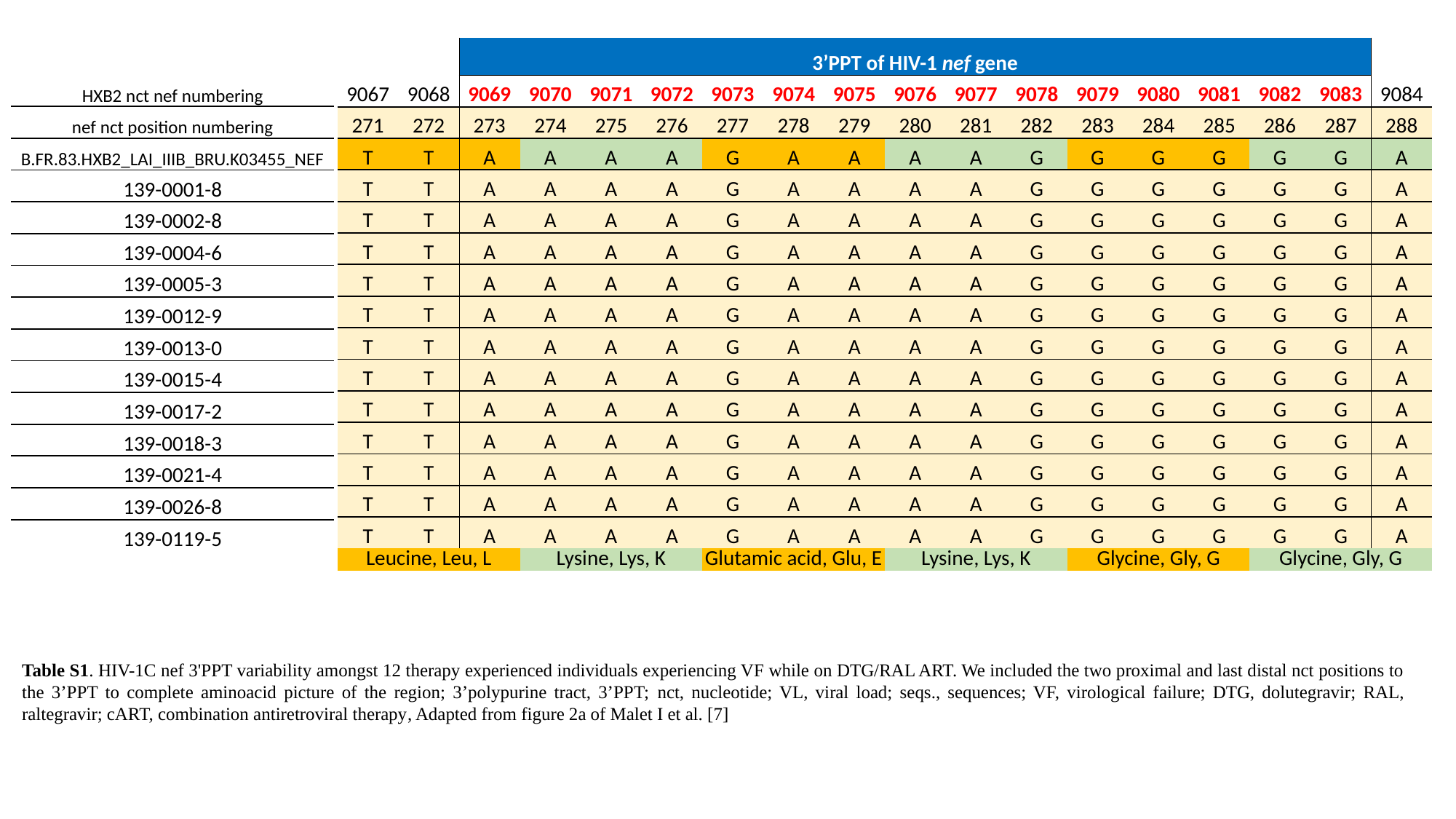

| | | 3’PPT of HIV-1 nef gene | | | | | | | | | | | | | | | |
| --- | --- | --- | --- | --- | --- | --- | --- | --- | --- | --- | --- | --- | --- | --- | --- | --- | --- |
| 9067 | 9068 | 9069 | 9070 | 9071 | 9072 | 9073 | 9074 | 9075 | 9076 | 9077 | 9078 | 9079 | 9080 | 9081 | 9082 | 9083 | 9084 |
| 271 | 272 | 273 | 274 | 275 | 276 | 277 | 278 | 279 | 280 | 281 | 282 | 283 | 284 | 285 | 286 | 287 | 288 |
| T | T | A | A | A | A | G | A | A | A | A | G | G | G | G | G | G | A |
| T | T | A | A | A | A | G | A | A | A | A | G | G | G | G | G | G | A |
| T | T | A | A | A | A | G | A | A | A | A | G | G | G | G | G | G | A |
| T | T | A | A | A | A | G | A | A | A | A | G | G | G | G | G | G | A |
| T | T | A | A | A | A | G | A | A | A | A | G | G | G | G | G | G | A |
| T | T | A | A | A | A | G | A | A | A | A | G | G | G | G | G | G | A |
| T | T | A | A | A | A | G | A | A | A | A | G | G | G | G | G | G | A |
| T | T | A | A | A | A | G | A | A | A | A | G | G | G | G | G | G | A |
| T | T | A | A | A | A | G | A | A | A | A | G | G | G | G | G | G | A |
| T | T | A | A | A | A | G | A | A | A | A | G | G | G | G | G | G | A |
| T | T | A | A | A | A | G | A | A | A | A | G | G | G | G | G | G | A |
| T | T | A | A | A | A | G | A | A | A | A | G | G | G | G | G | G | A |
| T | T | A | A | A | A | G | A | A | A | A | G | G | G | G | G | G | A |
| Leucine, Leu, L | | | Lysine, Lys, K | | | Glutamic acid, Glu, E | | | Lysine, Lys, K | | | Glycine, Gly, G | | | Glycine, Gly, G | | |
| HXB2 nct nef numbering |
| --- |
| nef nct position numbering |
| B.FR.83.HXB2\_LAI\_IIIB\_BRU.K03455\_NEF |
| 139-0001-8 |
| 139-0002-8 |
| 139-0004-6 |
| 139-0005-3 |
| 139-0012-9 |
| 139-0013-0 |
| 139-0015-4 |
| 139-0017-2 |
| 139-0018-3 |
| 139-0021-4 |
| 139-0026-8 |
| 139-0119-5 |
Table S1. HIV-1C nef 3'PPT variability amongst 12 therapy experienced individuals experiencing VF while on DTG/RAL ART. We included the two proximal and last distal nct positions to the 3’PPT to complete aminoacid picture of the region; 3’polypurine tract, 3’PPT; nct, nucleotide; VL, viral load; seqs., sequences; VF, virological failure; DTG, dolutegravir; RAL, raltegravir; cART, combination antiretroviral therapy, Adapted from figure 2a of Malet I et al. [7]

## Slide 2
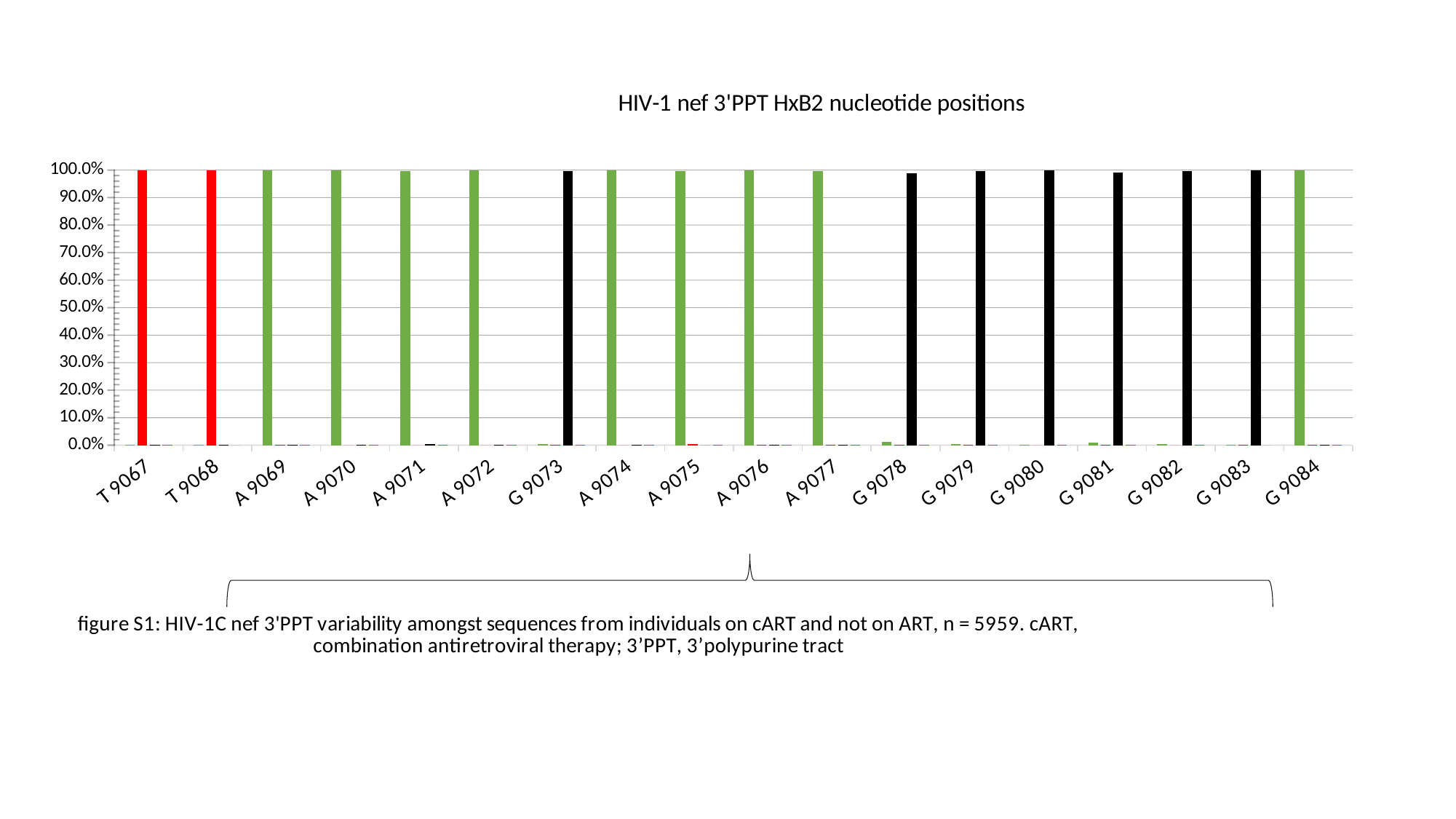

### Chart: HIV-1 nef 3'PPT HxB2 nucleotide positions
| Category | A | T | G | C |
|---|---|---|---|---|
| T 9067 | 0.00066711140760507 | 0.9986657771847899 | 0.0001667778519012675 | 0.0005003335557038026 |
| T 9068 | 0.0001667778519012675 | 0.9996664442961974 | 0.0001667778519012675 | 0.0 |
| A 9069 | 0.9993327773144287 | 0.00016680567139282736 | 0.0003336113427856547 | 0.00016680567139282736 |
| A 9070 | 0.9994996664442962 | 0.0 | 0.000333555703802535 | 0.0001667778519012675 |
| A 9071 | 0.9971652492913123 | 0.0 | 0.0026680006670001667 | 0.00016675004168751042 |
| A 9072 | 0.9988329443147715 | 0.0 | 0.0005001667222407469 | 0.0006668889629876625 |
| G 9073 | 0.0036672778796466078 | 0.0001666944490748458 | 0.995665944324054 | 0.0005000833472245374 |
| A 9074 | 0.9988331388564761 | 0.0 | 0.0003333888981496916 | 0.000833472245374229 |
| A 9075 | 0.996 | 0.0026666666666666666 | 0.0 | 0.0013333333333333333 |
| A 9076 | 0.9983333333333333 | 0.00016666666666666666 | 0.0008333333333333334 | 0.0006666666666666666 |
| A 9077 | 0.9975 | 0.0003333333333333333 | 0.0015 | 0.0006666666666666666 |
| G 9078 | 0.010668444740790132 | 0.0015002500416736123 | 0.9873312218703117 | 0.0005000833472245374 |
| G 9079 | 0.00383461153717906 | 0.00016672224074691563 | 0.9958319439813271 | 0.00016672224074691563 |
| G 9080 | 0.002001000500250125 | 0.0 | 0.9978322494580624 | 0.00016675004168751042 |
| G 9081 | 0.008501416902817137 | 0.0001666944490748458 | 0.9909984997499584 | 0.0003333888981496916 |
| G 9082 | 0.0026666666666666666 | 0.0 | 0.9971666666666666 | 0.00016666666666666666 |
| G 9083 | 0.0008333333333333334 | 0.0003333333333333333 | 0.9988333333333334 | 0.0 |
| G 9084 | 0.999 | 0.0003333333333333333 | 0.00016666666666666666 | 0.0005 |

## Slide 3
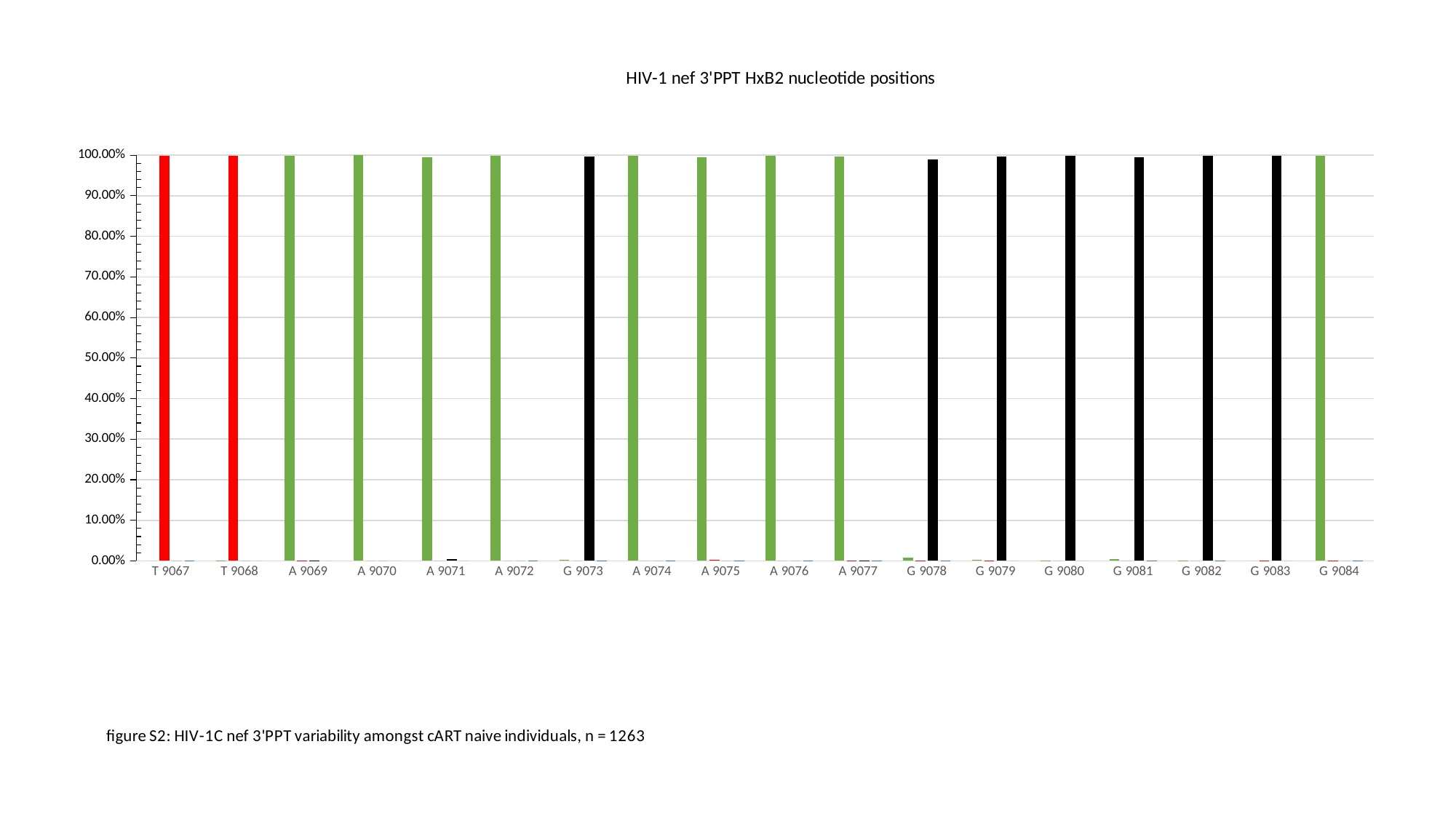

### Chart: HIV-1 nef 3'PPT HxB2 nucleotide positions
| Category | A | T | G | C |
|---|---|---|---|---|
| T 9067 | 0.0 | 0.9984114376489277 | 0.0 | 0.0015885623510722795 |
| T 9068 | 0.0007942811755361397 | 0.9992057188244639 | 0.0 | 0.0 |
| A 9069 | 0.9984114376489277 | 0.0007942811755361397 | 0.0007942811755361397 | 0.0 |
| A 9070 | 1.0 | 0.0 | 0.0 | 0.0 |
| A 9071 | 0.9952380952380953 | 0.0 | 0.004761904761904762 | 0.0 |
| A 9072 | 0.9992063492063492 | 0.0 | 0.0 | 0.0007936507936507937 |
| G 9073 | 0.002380952380952381 | 0.0 | 0.9968253968253968 | 0.0007936507936507937 |
| A 9074 | 0.9992063492063492 | 0.0 | 0.0 | 0.0007936507936507937 |
| A 9075 | 0.9952380952380953 | 0.0031746031746031746 | 0.0 | 0.0015873015873015873 |
| A 9076 | 0.9992063492063492 | 0.0 | 0.0 | 0.0007936507936507937 |
| A 9077 | 0.9968253968253968 | 0.0007936507936507937 | 0.0007936507936507937 | 0.0015873015873015873 |
| G 9078 | 0.007936507936507936 | 0.0015873015873015873 | 0.9888888888888889 | 0.0015873015873015873 |
| G 9079 | 0.002380952380952381 | 0.0007936507936507937 | 0.9968253968253968 | 0.0 |
| G 9080 | 0.0007942811755361397 | 0.0 | 0.9992057188244639 | 0.0 |
| G 9081 | 0.004761904761904762 | 0.0 | 0.9944444444444445 | 0.0007936507936507937 |
| G 9082 | 0.0015873015873015873 | 0.0 | 0.9976190476190476 | 0.0007936507936507937 |
| G 9083 | 0.0 | 0.0007936507936507937 | 0.9992063492063492 | 0.0 |
| G 9084 | 0.9984126984126984 | 0.0007936507936507937 | 0.0 | 0.0007936507936507937 |

## Slide 4
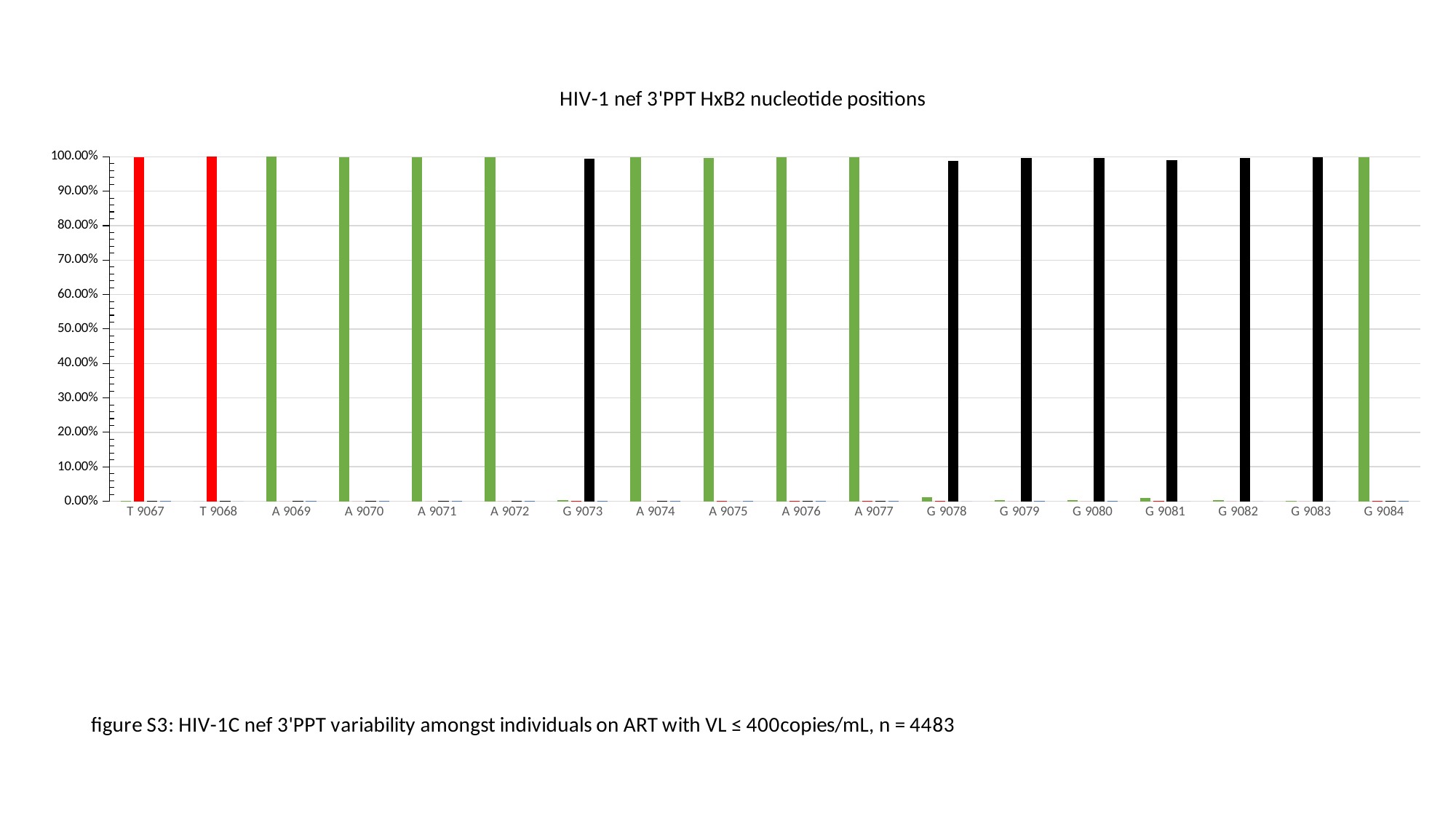

### Chart: HIV-1 nef 3'PPT HxB2 nucleotide positions
| Category | A | T | G | C |
|---|---|---|---|---|
| T 9067 | 0.0008934554389099843 | 0.9986598168416351 | 0.00022336385972749609 | 0.00022336385972749609 |
| T 9068 | 0.0 | 0.9997766361402725 | 0.00022336385972749609 | 0.0 |
| A 9069 | 0.9995531724754245 | 0.0 | 0.00022341376228775692 | 0.00022341376228775692 |
| A 9070 | 0.9993299084208175 | 0.0 | 0.00044672771945499217 | 0.00022336385972749609 |
| A 9071 | 0.997766361402725 | 0.0 | 0.0020102747375474648 | 0.00022336385972749609 |
| A 9072 | 0.9986601161232693 | 0.0 | 0.0006699419383653417 | 0.0006699419383653417 |
| G 9073 | 0.004242018307657959 | 0.00022326412145568208 | 0.995088189327975 | 0.00044652824291136416 |
| A 9074 | 0.9988834301027244 | 0.0 | 0.00022331397945511388 | 0.0008932559178204555 |
| A 9075 | 0.9966510381781648 | 0.0022326412145568207 | 0.0 | 0.0011163206072784104 |
| A 9076 | 0.9982138870283546 | 0.00022326412145568208 | 0.0008930564858227283 | 0.0006697923643670462 |
| A 9077 | 0.9979906229068989 | 0.00022326412145568208 | 0.0015628488501897744 | 0.00022326412145568208 |
| G 9078 | 0.011389012952210809 | 0.0015631978561857973 | 0.9870477891916034 | 0.0 |
| G 9079 | 0.004243913334822426 | 0.0 | 0.9955327228054501 | 0.00022336385972749609 |
| G 9080 | 0.002457002457002457 | 0.0 | 0.99731963368327 | 0.00022336385972749609 |
| G 9081 | 0.009381282108554836 | 0.00022336385972749609 | 0.9903953540317176 | 0.0 |
| G 9082 | 0.0029030817329164806 | 0.0 | 0.9970969182670835 | 0.0 |
| G 9083 | 0.0011165698972755694 | 0.0 | 0.9988834301027244 | 0.0 |
| G 9084 | 0.9993300580616347 | 0.00022331397945511388 | 0.00022331397945511388 | 0.00022331397945511388 |

## Slide 5
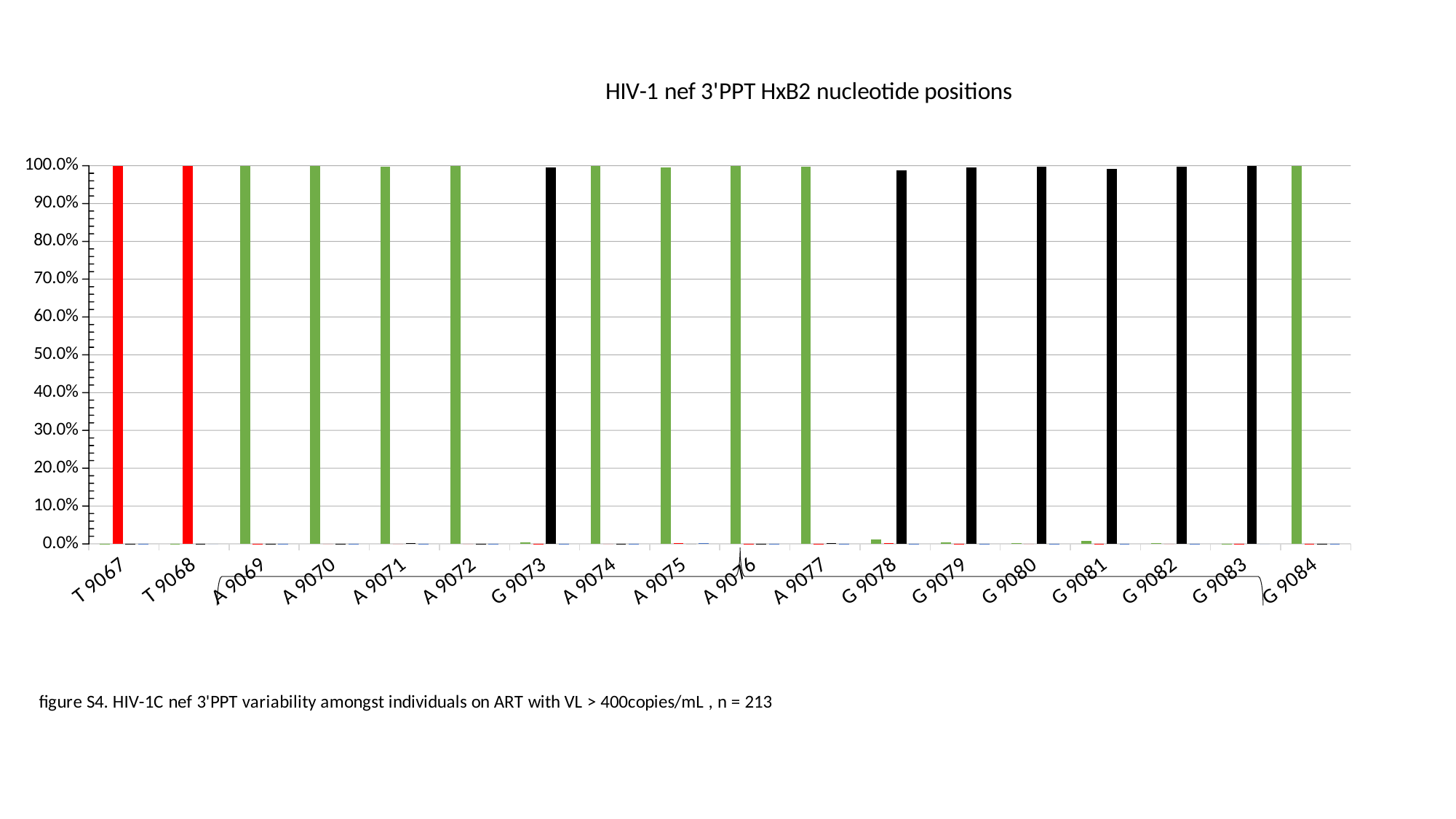

### Chart: HIV-1 nef 3'PPT HxB2 nucleotide positions
| Category | A | T | G | C |
|---|---|---|---|---|
| T 9067 | 0.00066711140760507 | 0.9986657771847899 | 0.0001667778519012675 | 0.0005003335557038026 |
| T 9068 | 0.0001667778519012675 | 0.9996664442961974 | 0.0001667778519012675 | 0.0 |
| A 9069 | 0.9993327773144287 | 0.00016680567139282736 | 0.0003336113427856547 | 0.00016680567139282736 |
| A 9070 | 0.9994996664442962 | 0.0 | 0.000333555703802535 | 0.0001667778519012675 |
| A 9071 | 0.9971652492913123 | 0.0 | 0.0026680006670001667 | 0.00016675004168751042 |
| A 9072 | 0.9988329443147715 | 0.0 | 0.0005001667222407469 | 0.0006668889629876625 |
| G 9073 | 0.0036672778796466078 | 0.0001666944490748458 | 0.995665944324054 | 0.0005000833472245374 |
| A 9074 | 0.9988331388564761 | 0.0 | 0.0003333888981496916 | 0.000833472245374229 |
| A 9075 | 0.996 | 0.0026666666666666666 | 0.0 | 0.0013333333333333333 |
| A 9076 | 0.9983333333333333 | 0.00016666666666666666 | 0.0008333333333333334 | 0.0006666666666666666 |
| A 9077 | 0.9975 | 0.0003333333333333333 | 0.0015 | 0.0006666666666666666 |
| G 9078 | 0.010668444740790132 | 0.0015002500416736123 | 0.9873312218703117 | 0.0005000833472245374 |
| G 9079 | 0.00383461153717906 | 0.00016672224074691563 | 0.9958319439813271 | 0.00016672224074691563 |
| G 9080 | 0.002001000500250125 | 0.0 | 0.9978322494580624 | 0.00016675004168751042 |
| G 9081 | 0.008501416902817137 | 0.0001666944490748458 | 0.9909984997499584 | 0.0003333888981496916 |
| G 9082 | 0.0026666666666666666 | 0.0 | 0.9971666666666666 | 0.00016666666666666666 |
| G 9083 | 0.0008333333333333334 | 0.0003333333333333333 | 0.9988333333333334 | 0.0 |
| G 9084 | 0.999 | 0.0003333333333333333 | 0.00016666666666666666 | 0.0005 |

## Slide 6
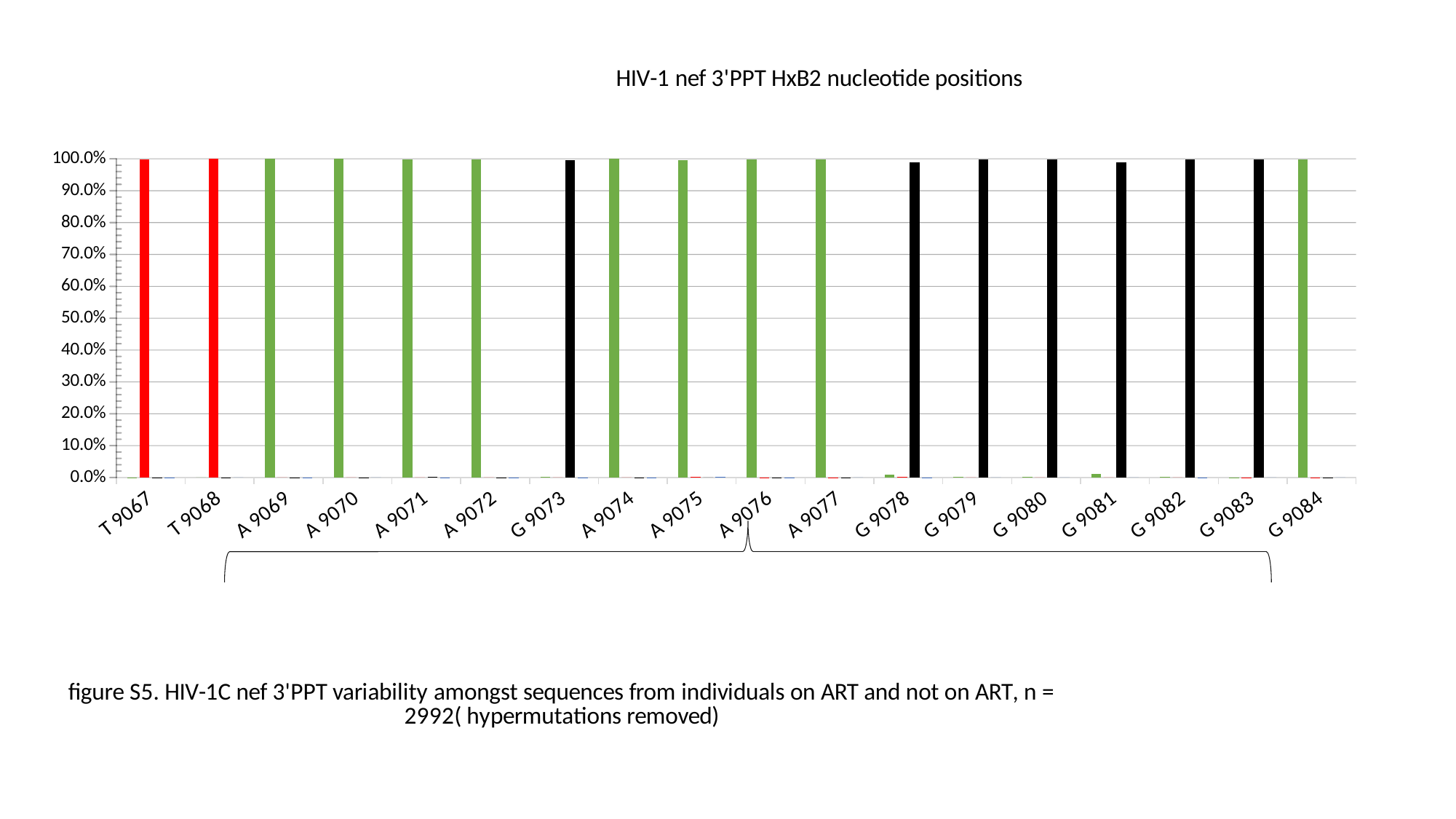

### Chart: HIV-1 nef 3'PPT HxB2 nucleotide positions
| Category | A | T | G | C |
|---|---|---|---|---|
| T 9067 | 0.0003348961821835231 | 0.9986604152712659 | 0.0003348961821835231 | 0.0006697923643670462 |
| T 9068 | 0.0 | 0.9996651038178165 | 0.0003348961821835231 | 0.0 |
| A 9069 | 0.999330207635633 | 0.0 | 0.0003348961821835231 | 0.0003348961821835231 |
| A 9070 | 0.9996651038178165 | 0.0 | 0.0003348961821835231 | 0.0 |
| A 9071 | 0.9986604152712659 | 0.0 | 0.0010046885465505692 | 0.0003348961821835231 |
| A 9072 | 0.9989953114534494 | 0.0 | 0.0003348961821835231 | 0.0006697923643670462 |
| G 9073 | 0.003013056578506863 | 0.0 | 0.9966521593572146 | 0.00033478406427854036 |
| A 9074 | 0.999330207635633 | 0.0 | 0.0003348961821835231 | 0.0003348961821835231 |
| A 9075 | 0.9963173752929361 | 0.002678272514228323 | 0.0 | 0.001004352192835621 |
| A 9076 | 0.9986608637428859 | 0.00033478406427854036 | 0.00033478406427854036 | 0.0006695681285570807 |
| A 9077 | 0.9989956478071644 | 0.00033478406427854036 | 0.0006695681285570807 | 0.0 |
| G 9078 | 0.009377093101138647 | 0.0013395847287340924 | 0.9889484259879437 | 0.0003348961821835231 |
| G 9079 | 0.0026791694574681848 | 0.0 | 0.9973208305425318 | 0.0 |
| G 9080 | 0.0023450586264656616 | 0.0 | 0.9976549413735344 | 0.0 |
| G 9081 | 0.010716677829872739 | 0.0 | 0.9892833221701273 | 0.0 |
| G 9082 | 0.002008704385671242 | 0.0 | 0.9976565115500502 | 0.00033478406427854036 |
| G 9083 | 0.0006695681285570807 | 0.00033478406427854036 | 0.9989956478071644 | 0.0 |
| G 9084 | 0.9989956478071644 | 0.0006695681285570807 | 0.00033478406427854036 | 0.0 |

## Slide 7
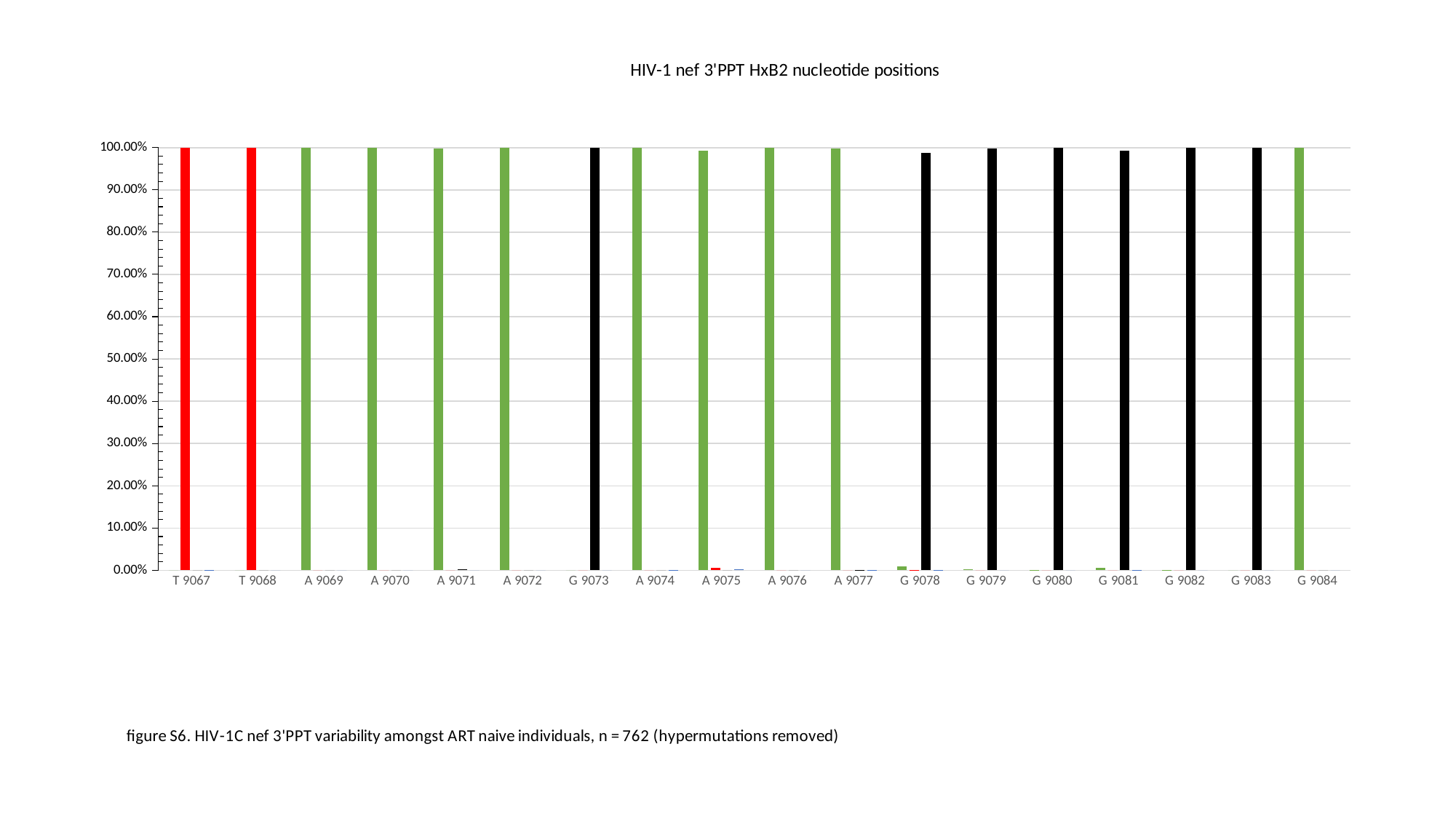

### Chart: HIV-1 nef 3'PPT HxB2 nucleotide positions
| Category | A | T | G | C |
|---|---|---|---|---|
| T 9067 | 0.0 | 0.9986859395532195 | 0.0 | 0.001314060446780552 |
| T 9068 | 0.0 | 1.0 | 0.0 | 0.0 |
| A 9069 | 1.0 | 0.0 | 0.0 | 0.0 |
| A 9070 | 1.0 | 0.0 | 0.0 | 0.0 |
| A 9071 | 0.9973753280839895 | 0.0 | 0.0026246719160104987 | 0.0 |
| A 9072 | 1.0 | 0.0 | 0.0 | 0.0 |
| G 9073 | 0.0 | 0.0 | 1.0 | 0.0 |
| A 9074 | 0.9986876640419947 | 0.0 | 0.0 | 0.0013123359580052493 |
| A 9075 | 0.9921259842519685 | 0.005249343832020997 | 0.0 | 0.0026246719160104987 |
| A 9076 | 1.0 | 0.0 | 0.0 | 0.0 |
| A 9077 | 0.9973718791064389 | 0.0 | 0.001314060446780552 | 0.001314060446780552 |
| G 9078 | 0.009198423127463863 | 0.001314060446780552 | 0.988173455978975 | 0.001314060446780552 |
| G 9079 | 0.002628120893561104 | 0.0 | 0.9973718791064389 | 0.0 |
| G 9080 | 0.001314060446780552 | 0.0 | 0.9986859395532195 | 0.0 |
| G 9081 | 0.006570302233902759 | 0.0 | 0.9921156373193167 | 0.001314060446780552 |
| G 9082 | 0.001314060446780552 | 0.0 | 0.9986859395532195 | 0.0 |
| G 9083 | 0.0 | 0.0 | 1.0 | 0.0 |
| G 9084 | 1.0 | 0.0 | 0.0 | 0.0 |

## Slide 8
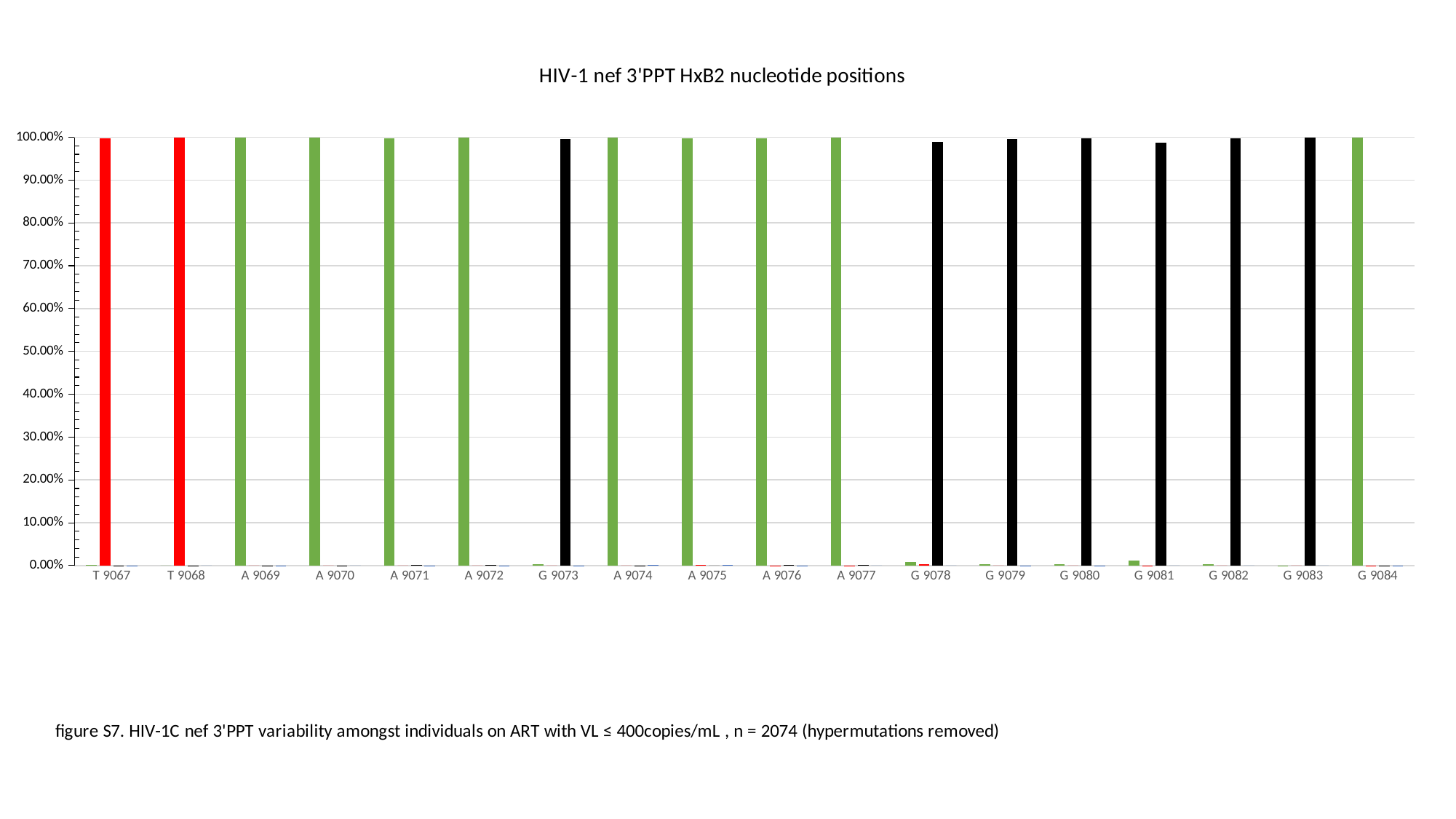

### Chart: HIV-1 nef 3'PPT HxB2 nucleotide positions
| Category | A | T | G | C |
|---|---|---|---|---|
| T 9067 | 0.0014478764478764478 | 0.9975868725868726 | 0.00048262548262548264 | 0.00048262548262548264 |
| T 9068 | 0.0 | 0.9995173745173745 | 0.00048262548262548264 | 0.0 |
| A 9069 | 0.999034749034749 | 0.0 | 0.00048262548262548264 | 0.00048262548262548264 |
| A 9070 | 0.9995173745173745 | 0.0 | 0.00048262548262548264 | 0.0 |
| A 9071 | 0.9980694980694981 | 0.0 | 0.0014478764478764478 | 0.00048262548262548264 |
| A 9072 | 0.9985521235521235 | 0.0 | 0.0009652509652509653 | 0.00048262548262548264 |
| G 9073 | 0.003376748673420164 | 0.0 | 0.9961408586589484 | 0.000482392667631452 |
| A 9074 | 0.9985521235521235 | 0.0 | 0.00048262548262548264 | 0.0009652509652509653 |
| A 9075 | 0.9966232513265798 | 0.001929570670525808 | 0.0 | 0.001447178002894356 |
| A 9076 | 0.9980704293294742 | 0.000482392667631452 | 0.000964785335262904 | 0.000482392667631452 |
| A 9077 | 0.9985528219971056 | 0.000482392667631452 | 0.000964785335262904 | 0.0 |
| G 9078 | 0.008204633204633204 | 0.002413127413127413 | 0.9893822393822393 | 0.0 |
| G 9079 | 0.0033783783783783786 | 0.0 | 0.9961389961389961 | 0.00048262548262548264 |
| G 9080 | 0.002413127413127413 | 0.0 | 0.997104247104247 | 0.00048262548262548264 |
| G 9081 | 0.012065637065637066 | 0.00048262548262548264 | 0.9874517374517374 | 0.0 |
| G 9082 | 0.002894356005788712 | 0.0 | 0.9971056439942113 | 0.0 |
| G 9083 | 0.000482392667631452 | 0.0 | 0.9995176073323685 | 0.0 |
| G 9084 | 0.9985528219971056 | 0.000482392667631452 | 0.000482392667631452 | 0.000482392667631452 |

## Slide 9
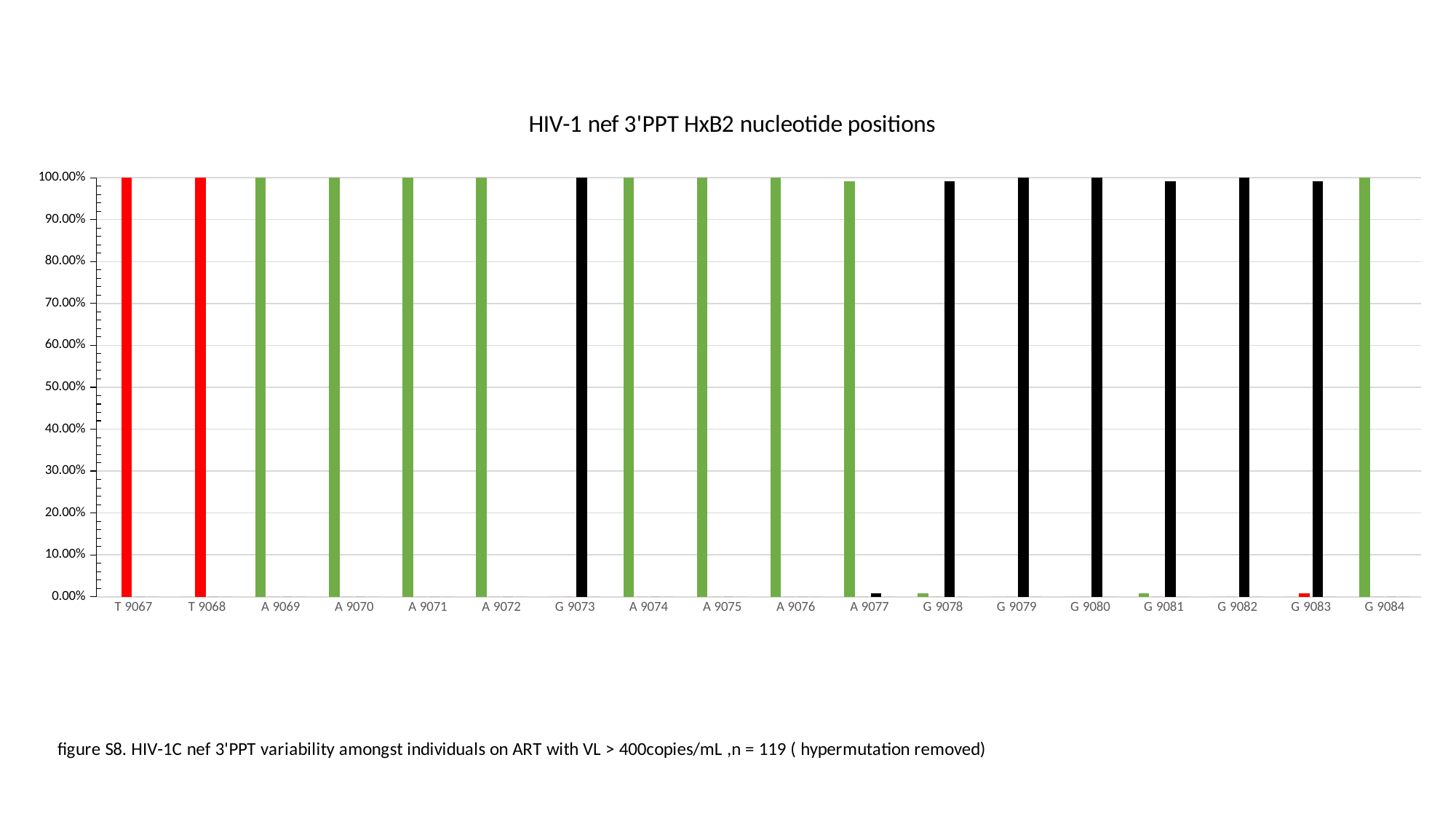

### Chart: HIV-1 nef 3'PPT HxB2 nucleotide positions
| Category | A | T | G | C |
|---|---|---|---|---|
| T 9067 | 0.0 | 1.0 | 0.0 | 0.0 |
| T 9068 | 0.0 | 1.0 | 0.0 | 0.0 |
| A 9069 | 1.0 | 0.0 | 0.0 | 0.0 |
| A 9070 | 1.0 | 0.0 | 0.0 | 0.0 |
| A 9071 | 1.0 | 0.0 | 0.0 | 0.0 |
| A 9072 | 1.0 | 0.0 | 0.0 | 0.0 |
| G 9073 | 0.0 | 0.0 | 1.0 | 0.0 |
| A 9074 | 1.0 | 0.0 | 0.0 | 0.0 |
| A 9075 | 1.0 | 0.0 | 0.0 | 0.0 |
| A 9076 | 1.0 | 0.0 | 0.0 | 0.0 |
| A 9077 | 0.9915966386554622 | 0.0 | 0.008403361344537815 | 0.0 |
| G 9078 | 0.008403361344537815 | 0.0 | 0.9915966386554622 | 0.0 |
| G 9079 | 0.0 | 0.0 | 1.0 | 0.0 |
| G 9080 | 0.0 | 0.0 | 1.0 | 0.0 |
| G 9081 | 0.008333333333333333 | 0.0 | 0.9916666666666667 | 0.0 |
| G 9082 | 0.0 | 0.0 | 1.0 | 0.0 |
| G 9083 | 0.0 | 0.008333333333333333 | 0.9916666666666667 | 0.0 |
| G 9084 | 1.0 | 0.0 | 0.0 | 0.0 |
